# Blue rubber bleb nevus syndrome with the complication of intussusception: A case report and literature review

**DOI:** 10.1097/MD.0000000000021199

**Published:** 2020-07-10

**Authors:** Zeming Hu, Xuan Lin, Jianing Zhong, Qingfang He, Qin Peng, Jianbo Xiao, Bin Chen, Jianhong Zhang

**Affiliations:** a Department of General Surgery, The First Affiliated Hospital of Gannan Medical University, Ganzhou, Jiangxi Province, China; b Key Laboratory of Prevention and Treatment of Cardiovascular and Cerebrovascular Diseases, Ministry of Education, Gannan Medical University, Ganzhou, Jiangxi Province, China; c Department of Pathology, The First Affiliated Hospital of Gannan Medical University, Ganzhou, Jiangxi Province, China.

**Keywords:** anemia, blue rubber bleb nevus syndrome, gastrointestinal bleeding, intussusception

## Abstract

**Rationale:**

Blue rubber bleb nevus syndrome (BRBNS) is an extremely rare disorder characterized by multifocal venous malformations involving various organs such as the skin and gastrointestinal tract. Severe complications of BRBNS, such as intussusception, volvulus, and intestinal infarction are rarer and require surgery. This report describes a 33-year-old male of BRBNS complicated with intussusception that was successfully diagnosed and treated with surgery.

**Patient concerns:**

A 33-year-old Chinese man presented with persistent, colicky pain accompanied by nausea, abdominal distension, and dizziness. The patient presented with sporadic bluish nodules on his skin involving his head, neck, thorax, abdomen, and planta pedis.

**Diagnoses:**

BRBNS with the complication of intussusception.

**Interventions:**

An emergency laparotomy was performed, and postoperative management included blood transfusions and oral iron supplementation for 2 weeks.

**Outcomes:**

The patient's postoperative course of hospitalization was uneventful. During the 4-month follow-up, the patient showed no signs of intussusception recurrence.

**Lessons:**

Patients diagnosed with BRBNS who present with acute abdominal pain and distension should raise suspicion for the presence of intussusception, which requires emergent surgical intervention.

## 1 Introduction

Blue rubber bleb nevus syndrome (BRBNS) is an extremely unusual disorder characterized by multifocal venous malformations involving various organs, with the affected organs mainly including the skin, gastrointestinal (GI) tract, and other visceral organs.^[1–4]^ The morbidity of BRBNS is very low and has been reported in more than 200 cases in an up-to-date MEDLINE search.^[5]^ The most frequent clinical manifestations of this syndrome are GI bleeding and secondary iron-deficiency anemia (IDA).^[6]^ Rarely, complications with BRBNS such as intussusception, volvulus, and intestinal infarction may occur during the disease history due to multiple GI hemangiomas.^[7]^

Herein, we present a case of BRBNS with acute intestinal intussusception and obstruction in a 33-year-old man who suffered from GI bleeding and severe anemia for years. The patient underwent emergent surgery on the first day of hospitalization.

## 2 Case presentation

A 33-year-old Chinese man was admitted to our hospital with a paroxysmal exacerbation of persistent, nonradiating colicky pain around the umbilical cord without obvious cause that had started the previous day. The patient had been unable to defecate and was experiencing nausea, abdominal distension, and dizziness. Since childhood, the patient had had multiple bluish cutaneous nodules throughout his entire body and had experienced recurrent hematochezia. However, the patient had no obvious symptoms except for pallor, fatigue, and melena, and he had never been hospitalized. In addition, the patient had no significant family history of this disorder.

**Figure 1:**
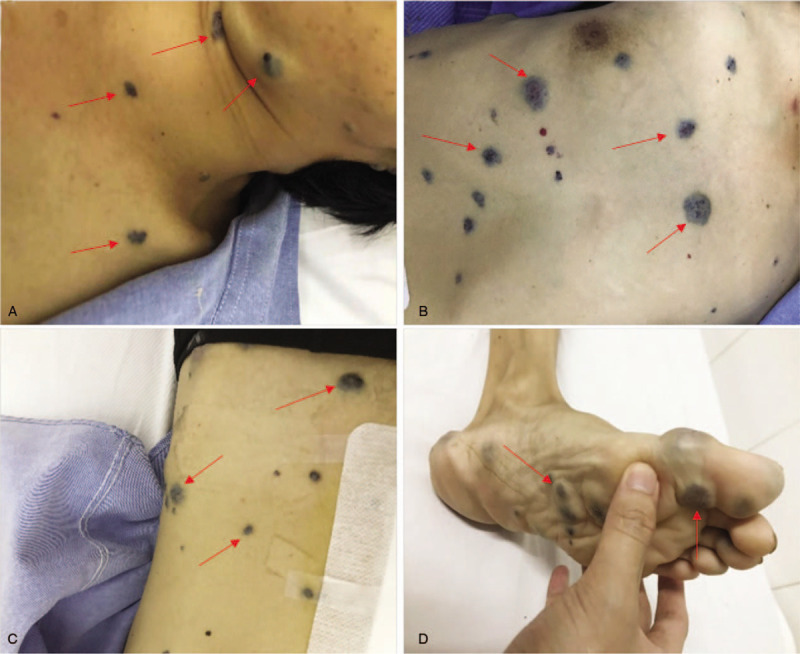
Representative images of skin lesions. Red arrows indicate multiple bluish nodules involving in the head and neck (A), thorax (B), abdomen (C,), and planta pedis (D).

On physical examination, the patient presented with sporadic, bluish, rubber-like hemangiomas on his entire body involving his head and neck (Fig. 1A), thorax (Fig. 1B), abdomen (Fig. 1C), and planta pedis (Fig. 1D). These compressible skin lesions ranged from 1 to 3 cm. The abdomen was flat and soft, and the patient complained of periumbilical tenderness.

The laboratory results revealed severe anemia characterized by small hypopigmented red blood cells and were as follows: hemoglobin, 54 g/L (normal range, 130–175 g/L); mean corpuscular volume, 66.5 fL (normal range 82–100 fL); mean corpuscular hemoglobin, 18.1 pg (normal range, 27–34 pg); mean corpuscular hemoglobin concentration, 272 g/L (normal range, 316–354 g/L). Red blood cell level was 3.49 × 10^12^/L (normal range, 4.3–5.8 × 10^12^/L), white blood cell level was 10.53 × 10^9^/L (normal range, 3.5–9.5 × 10^9^/L), and platelet counts were normal. The level of serum ferritin was 5.32 ng/mL (normal range, 30–400 ng/mL). Serum folic acid and vitamin B_12_ levels were normal. Bone marrow aspiration revealed normocellular marrow and depleted iron stores. Fecal occult blood test (FOBT) was positive. Taken together, these above results showed that the patient was most probably diagnosed as having IDA.

**Figure 2:**
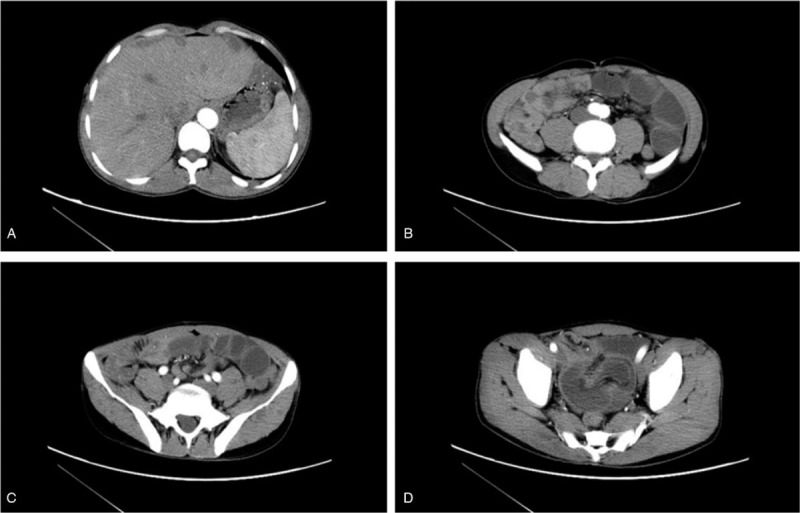
Images of abdominal and pelvic contrast-enhanced computed tomography (CT). (A) Probable hemangiomas or vascular malformations involving in the liver. (B–D) Typical signs of sleeve sheath and concentric circles revealed the presence of intussusception.

In addition, the abdominal and pelvic contrast-enhanced computed tomography (CT) scan indicated that multiple nodules and lamellar enhancement were observed in the liver parenchyma and the outer margin subcapsular area suspicious for hemangiomas or vascular malformations (Fig. 2A). The typical signs of sleeve sheath and concentric circles were observed in the pelvic intestinal canal suggesting the presence of intussusception and small bowel obstruction (Fig. 2B–D).

**Figure 3:**
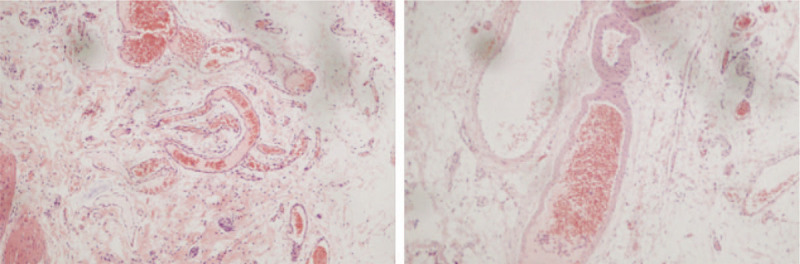
Histologic examination of intestinal lesions showed multiple thrombosed cavernous hemangiomas, which further confirmed the diagnosis of blue rubber bleb nevus syndrome.

On the basis of the clinical features and CT scans, the diagnosis of BRBNS was further confirmed, as the patient had not been previously diagnosed. Considering the ileus was severe, an emergency laparotomy was performed. During the surgical procedure, multiple hemangiomas of the colon and small intestine were observed. There were 3 segments of intussusceptions without necrosis in the ileum and a segment of intussusception that was unable to be reset at a distance from the ileocecal region and was excised. Subsequently, the pathological examination of resected lesions showed visible thrombosis, necrosis of the tissue wall, and inflammatory cell infiltration into the submucosa (Fig. 3). Postoperatively, the patient was managed with blood transfusions and oral iron supplementation for 2 weeks and he eventually recovered uneventfully. During the subsequent 4-month follow-up, the patient's hemoglobin increased to more than 110 g/L without any further treatment. Moreover, the patient has had no long-term complications after emergency surgery.

## 3 Discussion

BRBNS is a rare disease associated with congenital vascular malformations in the skin and the GI tract, with an incidence of approximately 1:14,000 births.^[2,8,9]^ The first report of the disease was described by Gascoyen in 1860,^[10]^ but the syndrome initially received its name (BRBNS, also called Bean syndrome) by Bean in 1958.^[11]^ Patients with BRBNS have a variety of venous malformations involving in various organs, including the liver, spleen, heart, eyes, and central nervous system.^[12]^ In the case presented here, lesions occurred in the liver without causing liver dysfunction; thus, we prescribed a regular, trimonthly review with CT scanning or ultrasonography.

The pathogenesis of this syndrome remains controversial. The incidence of BRBNS is similar in males and females, and is more common in Caucasians and less frequent in blacks and Asians, suggesting that prevalence may be related to geographic region.^[13,14]^ In addition, most cases are sporadic, but autosomal inheritance has been identified in a few familial cases, indicating that BRBNS may be associated with mutations in chromosome ^9^p.^[15]^ Recently, it has been reported that somatic mutation of *TIE2*, an endothelial cell tyrosine kinase receptor of angiopoietin, leads to this disease.^[13]^ Soblet et al^[16]^ analyzed a review of 17 patients with BRBNS and 6 patients with multifocal vascular malformations; the results showed that most patients with BRBNS have 2 somatic *TEK* mutations. The patient in our case had no significant family history of BRBNS, and he deferred genetic testing because of the limited detection technology for finding mutations related to BRBNS.

The clinical manifestations of BRBNS are variable depending on the involvement of different organs. The characteristic lesions are most frequently found on the skin and in the GI tract.^[3]^ Cutaneous lesions may occur mostly in infants and adolescents in the early stage, and are common in the upper limbs and especially the trunk as reported in the literature.^[4,17,18]^ Notably, cutaneous lesions associated with BRBNS are generally asymptomatic, although the number of lesions on the skin surface varies from several to more than 100.^[4,19]^ GI lesions can affect the mucosa from the oral cavity to the anus, but are more common in the small intestine and are prone to bleeding, usually leading to chronic IDA.^[2,20]^ In the present case, the patient had representative clinical features of the syndrome, such as skin lesions, GI bleeding, and severe IDA. One of the lessons learned from this case was that the GI lesions of BRBNS can also cause an abnormal intestinal peristaltic rhythm. The powerful peristalsis of the proximal intestine pushes the lesions concomitant with the intestine into the distal intestine, leading to the occurrence of volvulus, bowel obstruction, and intussusception. To our knowledge, there are only 9 cases of intussusception as a complication of BRBNS in the English literature, as identified from MEDLINE. As we summarized in Table 1, 8 of 10 patients (including our case) presented with typical cutaneous and GI lesions.^[21–28]^ Notably, one of the patients was pregnant and had BRBNS with the rare presentation of intussusception.^[19]^ According to our literature review, all of the patients had presented with the similar clinical characteristics of abdominal pain with or without GI bleeding. The literature showed that CT scanning and endoscopy are the main methods of detection for diagnosing BRBNS with intestinal intussusception effectively. All of the patients underwent successful laparotomy and had an uncomplicated postoperative course, excluding some cases that were lost to follow up. Similarly, our patient had an emergent laparotomy and had an uneventful postoperative course of hospitalization while being treated with iron supplementation and blood transfusions.

**Table 1:**
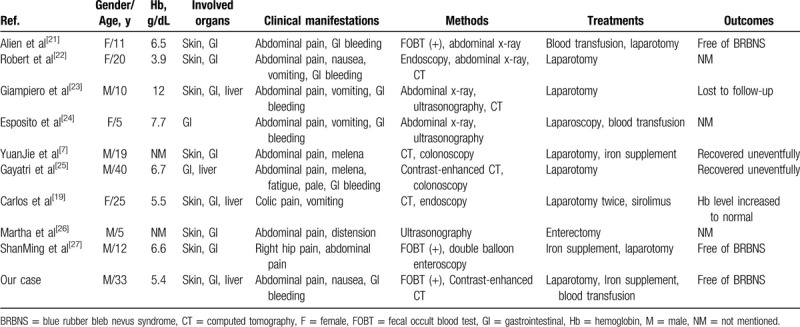
Cases of BRBNS with intussusception in MEDLINE search.

The diagnosis of BRBNS is predominantly based on the characteristic lesions of the skin and GI tract. Endoscopy is both the gold standard for diagnosing GI lesions and the treatment for these lesions.^[28]^ Abdominal radiographs may show calcification of visceral hemangioma lesions.^[23]^ The filling defect caused by the polypoid features of the hemangiomas can be observed by barium meal examination. CT scanning and magnetic resonance imaging (MRI) may be useful in locating lesions and defining the extent of disease.^[29,30]^ Capsule endoscopy is an effective method to evaluate small intestinal lesions on account of wide tolerability and acceptability by patients.^[10,31,32]^ In addition, FOBT is the main method to detect GI occult bleeding. In the case presented here, we combined the clinical manifestations of cutaneous lesions and routine blood analysis with emergent CT scan results to quickly establish the diagnosis of BRBNS.

Currently, there is no effective systematic treatment for BRBNS. Long-term iron supplementation is recommended for patients with BRBNS who have chronic blood loss and occasionally these patients need blood transfusions.^[14,29]^ Skin lesions generally do not require special treatment, and laser photocoagulation, sclerotherapy, or surgical resection options are only considered in terms of aesthetics and function.^[33]^ The treatment of GI lesions depends on the number, size, shape, location, and condition of the hemangiomas. Effective treatments that have been reported in the literature include sclerotherapy, angiographic embolization, electrocoagulation, laser coagulation, and surgical resection.^[34,35]^ Surgical resection is generally applicable to localized lesions, GI bleeding, intestinal ischemia, intussusception, and other complications, but its efficacy is still controversial because of the recurrence of lesions postoperatively. Drug therapy includes glucocorticoids, interferon-alpha, vincristine, and octreotide, which stabilize the prognosis of BRBNS and reduce GI bleeding by reducing vascular endothelial cell production and proliferation.^[34,36,37]^ Notably, sirolimus (Rapamycin) is a novel anti-angiogenic agent that has no adverse drug reactions and acts to reduce the size of venous malformations and control GI bleeding by inhibiting the mTOR signaling pathway.^[38]^

## 4 Conclusion

BRBNS with intussusception is a rare disorder characterized by multiple cutaneous and GI hemangiomas. Chronic IDA is a common complication of this disorder, whereas volvulus, intestinal obstruction, and intussusception are rarer. The present case suggests that clinicians should be on high alert for the occurrence of volvulus, ileus, and intussusception in patients with BRBNS presenting with acute abdominal pain.

## Acknowledgment

We especially thank the patient who participated in the case.

## Author contributions

**Conceptualization:** Jianhong Zhang.

**Data curation:** Zeming Hu, Xuan Lin, Jianing Zhong.

**Funding acquisition:** Bin Chen, Jianhong Zhang.

**Investigation:** Zeming Hu, Xuan Lin, Jianing Zhong.

**Methodology:** Qingfang He, Qin Peng, Jianbo Xiao.

**Resources:** Qingfang He, Qin Peng, Jianbo Xiao.

**Supervision:** Bin Chen, Jianhong Zhang.

**Writing – original draft:** Zeming Hu, Xuan Lin, Jianing Zhong.

**Writing – review & editing:** Bin Chen, Jianhong Zhang.
